# Aptamer-Based Microfluidic Assay for In-Field Detection of Salicylic Acid in *Botrytis cinerea*-Infected Strawberries

**DOI:** 10.3390/bios15050266

**Published:** 2025-04-22

**Authors:** Cristiana Domingues, Rafaela R. Rosa, Rodolfo G. Rodrigues, Ana Margarida Fortes, Virginia Chu, João Pedro Conde

**Affiliations:** 1 Instituto de Engenharia de Sistemas e Computadores–Microsistemas e Nanotecnologias (INESC-MN), Rua Alves Redol, 1000-029 Lisbon, Portugal; cdomingues@inesc-mn.pt (C.D.); rafaela.rosa@inesc-mn.pt (R.R.R.); rodolfo.rodrigues@inesc-mn.pt (R.G.R.); vchu@inesc-mn.pt (V.C.); 2 Instituto de Biossistemas e Ciências Integrativas (BioISI), Faculdade de Ciências de Lisboa, Universidade de Lisboa, 1749-016 Lisbon, Portugal; amfortes@ciencias.ulisboa.pt; 3 Department of Bioengineering, Instituto Superior Técnico, Avenida Rovisco Pais, 1049-001 Lisbon, Portugal

**Keywords:** microfluidics, salicylic acid, aptamer-based assay, optical sensor, agriculture

## Abstract

Rapid detection of plant infections is crucial for minimising crop loss and optimising management strategies, particularly in the context of climate change. While traditional diagnostic methods provide precise measurements of phytohormones such as salicylic acid (SA), a key regulator of plant defence responses, their reliance on bulky equipment and lengthy analysis times limits field applicability. This study presents a microfluidic-based aptamer assay for SA detection, enabling rapid and sensitive fluorescence-based readout from plant samples. A tailored sample pre-treatment protocol was developed and validated with real strawberry samples using HPLC measurements. The assay demonstrated a detection limit ranging from 10^−9^ to 10^−6^ mg/mL, within the relevant range for early infection diagnosis. The integration of the microfluidic platform with the optimised pre-treatment protocol offers a portable, cost-effective solution for on-site phytohormone analysis, providing a valuable tool for early infection detection and improved crop management.

## 1. Introduction

The increasing global population, projected to surpass 9.7 billion by 2050, requires a substantial rise in agricultural production, estimated at 60–110% according to the Food and Agriculture Organization (FAO) [1]. However, this goal is challenged by climate change, soil degradation, and pollution, which threaten crop yields and food security. Among these challenges, plant diseases pose a significant risk, with their rapid spread impacting both economic stability and environmental sustainability [2]. As climate conditions become more unpredictable, early detection of plant infections is crucial for timely intervention, preventing disease outbreaks, and minimising losses [3,4,5].

The traditional approach for pathogen detection is by directly detecting the pathogen’s genetic material through DNA amplification techniques, most commonly the polymerase chain reaction (PCR). PCR provides great sensitivity and specificity, and remains the gold standard; however, it involves complex sample preparation procedures, lengthy analysis times, and diverse laboratory equipment that cannot be taken to the field [6,7].

One promising approach for early pathogen detection is monitoring changes in phytohormone levels before visible infection symptoms appear. In particular, salicylic acid (SA) plays a key role in plant defence, as its concentration increases in response to biotic stress, such as fungal infections [8]. However, conventional detection methods, including High-Performance Liquid Chromatography (HPLC), while highly accurate, are impractical for field use due to bulky equipment, skilled operator requirements, and lengthy analysis times [1,9]. These limitations delay intervention, reducing the effectiveness of disease management, especially during rapidly spreading infections. Thus, there is an urgent need for portable, cost-effective point-of-care (PoC) diagnostic tools that enable real-time, in-field SA detection, particularly in resource-limited settings [10,11,12].

Despite the advantages of PoC methods for plant hormone analysis, their availability remains limited. Most existing detection techniques rely on laboratory-based approaches, which lack the portability required for field applications. Optical aptamer-based sensors integrated with microfluidic platforms have shown promise for detecting plant hormones such as abscisic acid (ABA), offering rapid and sensitive detection [9]. However, the absence of suitable sample pre-treatment protocols for complex plant matrices limits their practical implementation. Addressing this gap requires the development of streamlined, field-compatible diagnostic tools capable of integrating robust sample processing with highly sensitive detection mechanisms.

This study presents a novel microfluidic-based aptamer assay for SA detection in strawberry samples. SA (molar mass: 138.12 g/mol) plays a key role in plant defence, particularly in the systemic acquired resistance (SAR) pathway [13]. During infection, SA accumulates at the infection site, triggering protective mechanisms and limiting pathogen spread. SA levels vary among strawberry cultivars, and monitoring these fluctuations helps establish a baseline for plant health [8,14,15]. In strawberries, SA concentrations typically range from 10^−5^ to 10^−1^ mg/mL [9].

Strawberries are highly susceptible to fungal infections such as *Botrytis cinerea*, the causal agent of grey mould, which thrives in humid conditions and can cause substantial yield loss, reduced fruit quality, and shorter post-harvest shelf life [16]. Even at undetectable infection levels, grey mould can accelerate spoilage during transport and storage [17,18]. Previous studies have shown that SA levels increase in grapes infected with *Botrytis cinerea*, suggesting a broader role for SA in plant immune responses [12]. Monitoring SA fluctuations in strawberry plants could provide valuable insights into infection progression and improve disease management strategies.

Aptamer-based sensors offer a highly specific and sensitive alternative to conventional antibody-based assays. Aptamers, single-stranded DNA or RNA molecules selected through the Systematic Evolution of Ligands by Exponential enrichment (SELEX), exhibit strong binding affinities (picomolar to nanomolar range) and superior stability under various environmental conditions [19,20]. Unlike antibodies, aptamers can be synthesised against targets with low immunogenicity, ensuring high reproducibility and minimal batch-to-batch variability [21,22,23]. Their ability to undergo reversible denaturation allows for potential sensor reusability, making them well-suited for field applications [24].

Microfluidic technologies provide an ideal platform for phytohormone detection due to their precise fluid handling, controlled reaction environments, and enhanced assay kinetics [1,2,25,26]. By integrating an aptamer-based fluorescence assay within a microfluidic system, we enable rapid, sensitive, and field-deployable SA detection. In this study, we developed a tailored sample pre-treatment protocol for real strawberry samples and validated the assay using HPLC measurements. Our results demonstrate the potential of this microfluidic platform for real-time SA monitoring, offering a practical tool for early infection detection and improved agricultural disease management.

## 2. Materials and Methods

### 2.1. Fabrication of the Microfluidic Devices

The process for fabricating microfluidic devices with immobilised microbeads has been documented elsewhere [2]. The microchannel designs were created using AutoCAD 2024 software (version 24.3) (Autodesk, San Francisco, CA, USA). To create the aluminium hard masks, the design pattern was transferred to a Corning Eagle glass substrate with a layer of aluminium using direct-write laser lithography (Heidelberg DWLII, Heidelberg Instruments, Heidelberg, Germany), followed by etching with Gravure Aluminium Etchant (Microchemicals, Ulm, Germany). Since the microfluidic channels are made up of two different heights, two aluminium hard masks were fabricated. Using these hard masks, an SU-8 master mould was then manufactured using two different SU-8 photoresists, one for each height (SU-8 2015 for 20 µm, SU-8 50 for 100 µm layers, both from Microchem, Newton, MA, USA). The photoresist was spin-coated on top of a silicon wafer followed by exposure to UV light through the aluminium hard mask (UV KUB-2, KLOÉ, Saint-Mathieu-de-Tréviers, France) and then baked. Following the baking, the unexposed photoresist was removed with propylene glycol methyl ether acetate (PGMEA, Sigma-Aldrich, St. Louis, MO, USA). To fabricate the PDMS structures (polydimethylsiloxane), the curing agent and base were mixed in a ratio of 1:10 (*w*/*w*) and degassed. The mixture was then poured onto the SU-8 mould and covered with a flat poly (methyl methacrylate) (PMMA) frame and placed in the oven to cure at 70 °C for 90 min. Following this, the inlets and outlets of the structure were punctured, and the structure was sealed against a 500 µm-thick PDMS slab (fabricated under the same conditions) using oxygen plasma (Harrick Plasma, Ithaca, NY, USA).

### 2.2. Aptamer-Based Assay for SA Detection

#### 2.2.1. Reagents

**Figure 1:**
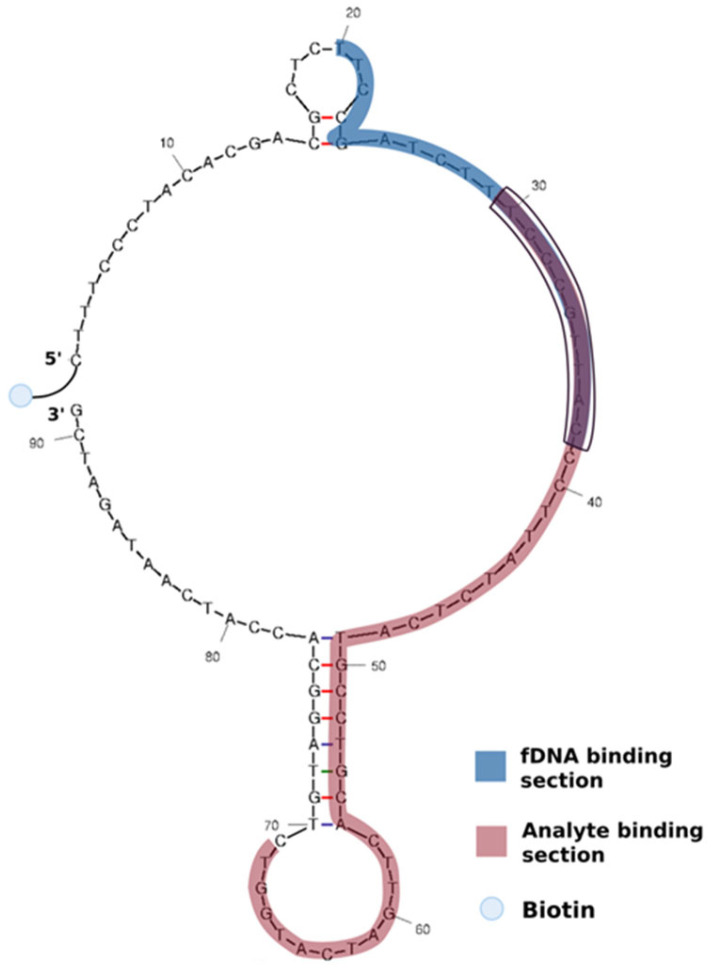
Schematic representation of the aptamer biotinylated at the 5′ end folding structure under buffer conditions. In blue is highlighted the fDNA binding section, and in red the analyte binding section, with the overlapping portion in purple. Schematic generated in mFold, considering the salt concentrations and pH conditions used in the test [27].

Streptavidin microbeads (45–90 µm diameter) were sourced from Merck (Alameda Fernão Lopes, Algés, Portugal), while Phosphate Buffered Solution (PBS) 10×, and TE buffer were obtained from Fisher Scientific (Waltham, MA, USA). The biotinylated SA aptamer (the sequence was obtained from Chen et al. [22], who performed the SELEX for this aptamer, represented in Figure 1) and fluorescently labelled DNA (fDNA) were provided by Integrated DNA Technologies (Coralville, IA, USA), and both were resuspended in TE buffer to a 100 µM concentration—Table 1. Sodium chloride (NaCl), magnesium chloride (MgCl₂), Tris(hydroxymethyl)aminomethane, and methanol (MeOH), anhydrous 99.8%, were purchased from Sigma-Aldrich (St. Louis, MO, USA). Lastly, Q-Sepharose microbeads (90 µm diameter) were obtained from Cytiva (Björkgatan, Uppsala, Sweden).

#### 2.2.2. Packing the Biomarker Detection Chamber

The streptavidin microbeads were introduced into the microfluidic structure using a pipette tip, and exerting a controlled negative pressure applied at both the inlet_1_ and outlet with a syringe pump (NE-4000, New Era Pumps, Farmingdale, NY, USA). The channel was firstly filled with PBS 1× by placing a pipette tip at inlet_2_, followed by a 20 µL solution of bead suspension at a flow rate of 5 µL/min, allowing the beads to accumulate inside the chamber. Once the beads were packed, inlet_2_ was closed with a 20-gauge metal plug, and the chamber was washed with PBS 1× at 15 µL/min. The structure was then submerged in deionised water (DI) and refrigerated overnight to remove any air bubbles that were introduced into the system during the packing process.

#### 2.2.3. Fluorescence-Based Aptamer for SA Detection

The aptamer-based assay was conducted using a microfluidic system containing a microcolumn packed with streptavidin microbeads, as described in Section 2.2.2, following a series of preparation, incubation, and detection steps. First, the aptamer solution was heated to 95 °C for 5 min to denature and unfold its structure, followed by rapid cooling in ice for 1 min to promote proper refolding into its active conformation. The analyte was then mixed with the aptamer and incubated outside the microfluidic channel for 30 min to allow sufficient binding before being introduced into the microfluidic system. The analyte–aptamer solution was then flowed through the microcolumn at 1 µL/min for 20 min, ensuring effective interaction with the microbeads. Following this, a fDNA probe was introduced at 1 µL/min for 5 min to enable signal detection. The fDNA sequence was designed to bind to a specific region of the aptamer, in such a way that it binds partially to the target biding section of the aptamer, and partially outside of this region, as seen in Figure 1. The target binding region, highlighted in red in Figure 1, was estimated from the results obtained by Chen et al. [24] through the SELEX process, where this region is changed throughout the cycles, until an aptamer specific to the target is obtained. This interaction allows for the detection of SA, given that the binding of SA to the aptamer influences the ability of the fDNA to bind to the aptamer as well. Lastly, to remove any unbound molecules and minimise background noise, the system was washed with SA buffer at 2.5 µL/min for 2 min.

### 2.3. Sample Collection and Preparation for SA Detection in Strawberries

**Figure 2:**
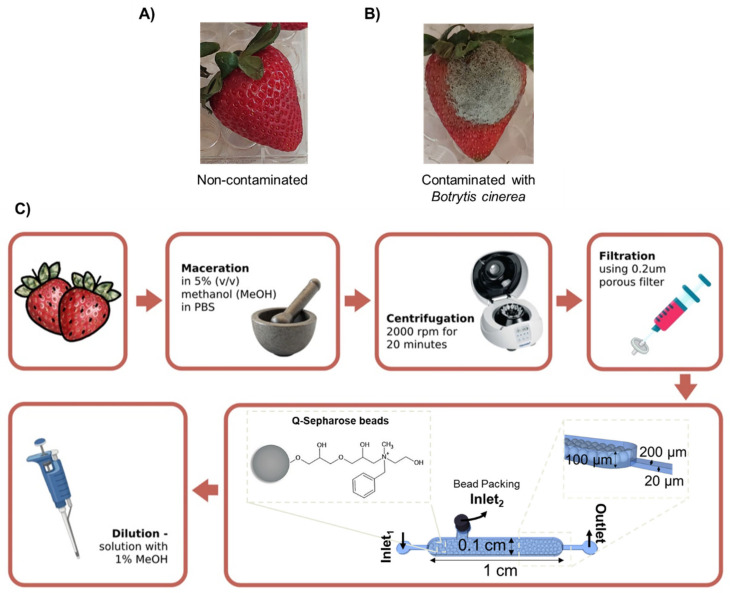
Photographs of supermarket-purchased strawberries and Botrytis cinerea-infected strawberries ((**A**) and (**B**), respectively), alongside a schematic representation of the sample treatment process (**C**)). The process includes maceration of the strawberries with 5% of MeOH, followed by centrifugation (2000 rpm, 20 min), filtration through a 0.2 µm pore filter, and a bead cleaning step within the microfluidic channel. The columns used for bead trapping were 1 cm long, 0.1 cm wide, and 100 µm high. Additionally, a smaller channel (200 µm wide, 20 µm high) was incorporated to capture Q- Sepharose microbeads larger than 20 µm in diameter.

To evaluate the sensitivity of the SA detection assay, strawberries were purchased from a local supermarket and stored at −80 °C until further analysis. These strawberries were selected because they were not expected to contain high levels of SA (Figure 2A), in contrast to infected strawberries, which typically have higher levels of SA. The strawberries were analysed using HPLC, which confirmed the absence of SA in the samples. Figure S1 in the Supplementary Materials displays the chromatograms obtained from HPLC.

To validate the developed method, strawberries infected with *Botrytis cinerea* (Figure 2B) were collected from a local farm. These were quickly frozen in liquid nitrogen, transported in dry ice to the laboratory, and kept at −80 °C until processing. The infected strawberry samples were analysed using HPLC, which confirmed the presence of salicylic acid (SA) at a concentration of 8 × 10^−3^ mg/mL. Figure S2 in the Supplementary Materials displays the calibration curve used to quantify the amount of SA in the infected strawberry sample.

The sample preparation, as shown in Figure 2C, begun by macerating the strawberries in a 5% methanol (MeOH) solution in PBS 1×, mixed at a 1:1 ratio (*w*/*v*) for 8 min. The mixture was then centrifuged at 2000 rpm for 20 min, and the pellet of cell debris at the bottom was discarded. To further purify the sample and remove large debris, the supernatant was filtered through a 0.2 µm nylon syringe filter. To remove any remaining contaminants not captured by the filter, the solution was flowed through Q-Sepharose beads packed in a microfluidic channel at 5 µL/min for 10 min. Lastly, the solution was diluted to achieve a 1% MeOH concentration in the sample, due to preliminary tests showing that a higher MeOH percentage hinders molecular recognition.

#### Packing of the Sample Treatment Chamber

The Q-Sepharose microbeads were inserted into the microchannel in a similar manner as described in Section 2.2.2. The microchannel was firstly filled with PBS 1× by placing a pipette tip at inlet_2_ and applying negative pressure with a syringe pump. Following this, a pipette with 30 µL bead suspension solution was placed at inlet_2_ (Figure 2) and a 12 µL/min flow rate was applied until the microchannel was fully packed. This was followed by washing with PBS 1× at 22 µL/min and sealing inlet_2_ with a 20-gauge metal plug.

### 2.4. Fluorescence Imaging and Data Analysis

The fluorescence signals for the experimental assay were collected using a Leica DMLM microscope, coupled with a DFC300FX digital camera and a CoolLED pE-300lite lamp for excitation. The imaging system was fitted with an I3 filter cube, which enabled excitation in the 450–490 nm range (blue) and captured fluorescence emission above 515 nm (green). Image analysis was conducted with ImageJ software 1.49 (NIH, USA), focusing solely on the green fluorescence channel. To quantify fluorescence intensity, the background signal was subtracted from a reference region outside the experimental area. All images used for SA quantification were acquired with a fixed exposure time of 1 s and a gain setting of 1× to maintain measurement consistency.

### 2.5. Quantification of SA Using Reverse-Phase HPLC

The concentration of SA in strawberry samples was measured using a Hitachi LaChrom HPLC system, which consisted of two pumps (L-7100), a UV detector (L-7400), a programmable autosampler (L-7250), and a data interface (Hitachi D-7000) for computer connectivity. A core-shell organo-silica LC column (5 µm EVO C18 100 Å, 250 × 4.6 mm) from Kinetex was employed for the analysis, with UV detection set at 206 nm. Chromatographic separation was performed in isocratic mode, using a mobile phase composed of sodium phosphate buffer (50 mM, pH 3.55, Sigma-Aldrich) and acetonitrile (VWR) in a 75:25 (*v*/*v*) ratio. The injection volume was 25 µL, with a flow rate of 1.2 mL/min, following the procedure described in reference [2]. Under these conditions, the retention time of SA was approximately 3.12 min.

## 3. Results

This study developed a microfluidic-based method for detecting salicylic acid (SA) in strawberries, leveraging SA as a biomarker for plant stress and pathogen response. SA plays a crucial role in plant defence, particularly against biotic stress, such as pathogen infections. The detection strategy utilises a biotinylated SA aptamer immobilised on streptavidin-coated microbeads, which are mechanically trapped within the microfluidic channel. The incorporation of microbeads enhances assay sensitivity and efficiency by increasing the surface area for aptamer immobilisation and minimising diffusion distances, thereby reducing assay time. In this approach, a fluorescently labelled DNA strand (fDNA) is designed to hybridise with a specific region of the aptamer. Upon SA binding, the aptamer undergoes a conformational change, increasing the accessibility of the fDNA binding site and leading to an enhanced fluorescence signal, enabling sensitive and specific SA detection.

### 3.1. SA Spiked in Binding Buffer

The first step in developing the assay was to determine the optimal concentrations of the SA aptamer and fDNA to ensure a functional and sensitive detection method. Figure S3 in the Supplementary Materials shows a summary of the optimisation steps. These concentrations were carefully optimised to generate a measurable fluorescence signal while maintaining responsiveness to varying SA levels. The fDNA concentration was set slightly higher than that of the aptamer to facilitate competitive binding. Excessive fDNA concentration would lead to no competition with SA for aptamer binding, reducing assay sensitivity, whereas too little fDNA would lead to indiscriminate binding, making it difficult to distinguish SA concentrations. After optimisation, the SA aptamer and fDNA concentrations were fixed at 0.3 µM and 0.4 µM, respectively.

**Figure 3:**
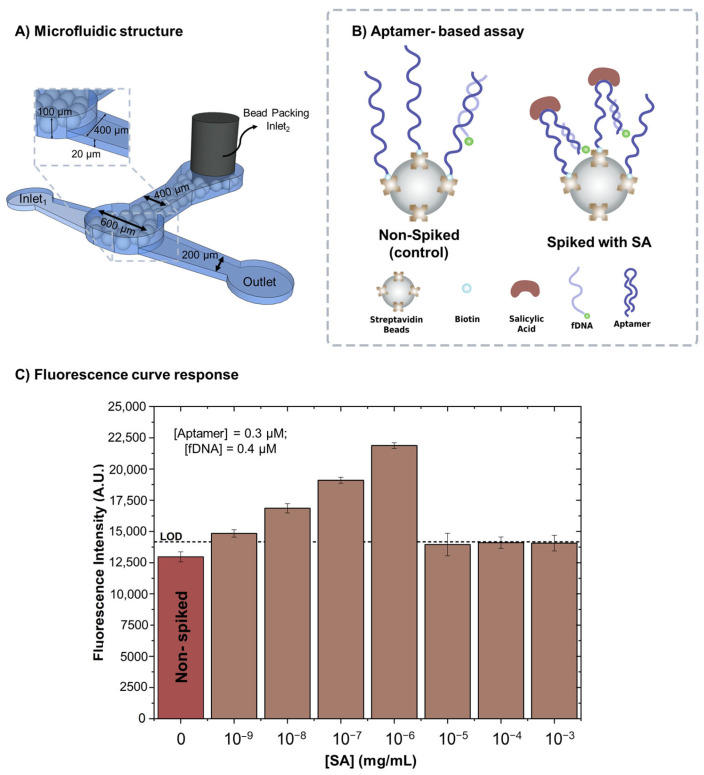
Aptamer-based assay for SA detection: (**A**) The microfluidic device for biomarker detection was designed with distinct channel heights to facilitate different functions within the system. A 100 µm-high channel was allocated specifically for bead packing, allowing efficient retention and arrangement of the streptavidin-coated microbeads (approximately 90 µm in diameter). Meanwhile, a 20 µm-high channel was used for the controlled injection of samples and reagents. The variation in channel heights played a crucial role in bead confinement, as the lower-height inlet and outlet channels acted as physical barriers, effectively preventing the passage of microbeads larger than 20 µm while allowing fluid flow; (**B**) the detection strategy involved immobilising SA aptamers onto streptavidin-coated microbeads, which were mechanically confined within the microfluidic channel. In this approach, the fDNA is designed to complement a specific region of the aptamer. When the analyte is present, the aptamer undergoes a conformational change that enhances the binding affinity of the fDNA; (**C**) the fluorescence response curve was generated for varying SA target concentrations, ranging from 10^⁻9^ mg/mL to 10^⁻3^ mg/mL. The excitation wavelength was set between 450 and 490 nm (blue). Error bars indicate the standard deviation (±) of four replicas.

After establishing the optimal concentrations of SA aptamer and fDNA, we evaluated the relationship between fluorescence intensity and varying SA concentrations in the binding buffer (20 mM Tris, pH 7.5, 5 mM MgCl₂, 137 mM NaCl) [9,24]. A series of SA solutions, diluted from an initial stock concentration of 5 mg/mL, were prepared to determine the minimum detectable SA concentration using the microfluidic system and aptamer-based assay, as shown in Figure 3. The fluorescence signal varied with SA concentration, reflecting the dynamic interactions between SA, fDNA, and the immobilised aptamer. Within the concentration range of 10^−9^ to 10^−6^ mg/mL, fluorescence intensity increased with higher SA concentrations, indicating that SA binding induces a conformational change in the aptamer, enhancing its affinity for fDNA. However, at higher SA concentrations (10^−5^ to 10^−3^ mg/mL), the fluorescence signal plateaued, similar to the non-spiked assay. This behaviour suggests that at higher SA concentrations, the analyte may form aggregates, rendering the aptamer binding site inaccessible, which prevents efficient capture and results in a reduced fluorescence signal, returning to baseline levels.

The selectivity of this detection method was confirmed using different analytes, ABA, and jasmonic acid (JA), for the highest detectable concentration, 10^−6^ mg/mL, showed no change from the non-spiked assay. These supporting data are in Figure S4 in the Supplementary Information.

### 3.2. SA Spiked in Treated Supermarket Strawberry Samples

**Figure 4:**
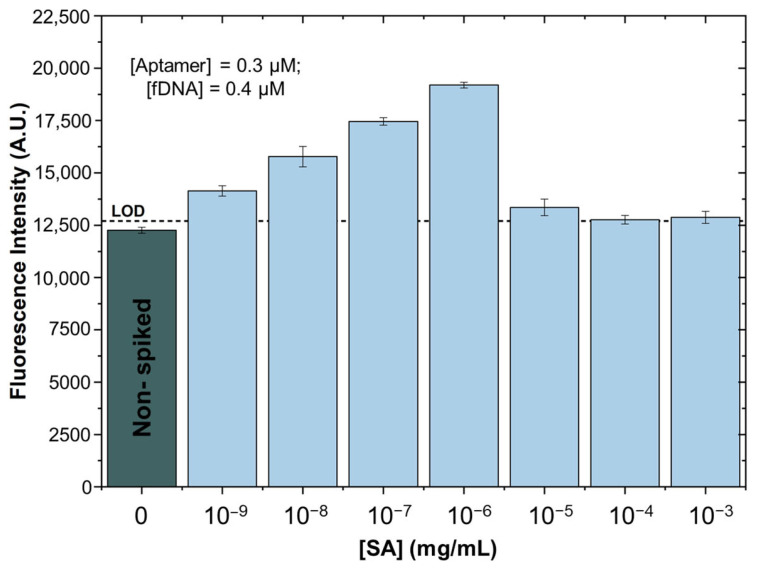
Fluorescence curve response in strawberries. The aptamer-based assay for detecting SA was conducted using strawberry samples spiked with SA, with concentrations varying from 10^−9^ to 10^−3^ mg/mL. Fluorescence was measured with an excitation wavelength between 450 and 490 nm (blue). Error bars indicate the standard deviation (±) of two replicas.

After validating the assay’s sensitivity under optimal conditions using SA in the binding buffer, we tested the method on strawberries purchased from a local supermarket, which were free from detectable SA (as detailed in Figure S1 of the Supplementary Materials). A specific sample preparation protocol was developed, as outlined in Figure 2 and described in Section 2.3. A critical step in this protocol was bead cleaning, which removed large particles that might have bypassed initial filtration and interfered with detection. Applying this procedure to strawberry samples, we conducted the microfluidic assay and collected fluorescence data (Figure 4), establishing a correlation between SA concentration and fluorescence intensity. The results indicated that the fluorescence response from the strawberry samples closely matched that of the binding buffer, confirming the effectiveness of the sample preparation in eliminating most interfering compounds. The slight reduction in fluorescence observed with strawberry samples, compared to buffer-only assays, is likely due to residual contaminants. With a validated method for detecting SA in the binding buffer and an effective preparation process for strawberry samples, we next analysed SA levels in infected strawberries, anticipating a natural increase in SA concentration during infection.

### 3.3. SA in Naturally Infected Strawberry Samples

To validate the SA detection method, we selected strawberry samples based on their expected SA levels, given SA’s key role in plant defence mechanisms. Infected strawberries naturally accumulate higher SA concentrations in response to pathogen attacks, while healthy strawberries typically have minimal SA levels. Prior to analysis, all samples underwent a standardised preparation process (outlined in Section 2.3) to ensure consistency and eliminate potential interferents. High-Performance Liquid Chromatography (HPLC) analysis confirmed significantly higher SA concentrations in infected strawberries. Quantification, detailed in Figure S2 of the Supplementary Materials, determined the SA concentration in these samples to be 8 × 10^−3^ mg/mL.

**Figure 5:**
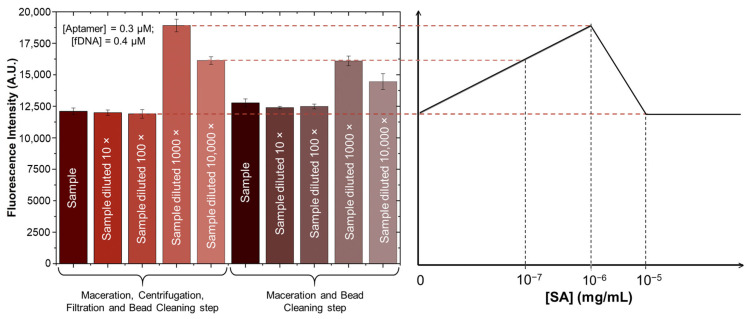
Validation of SA detection method in real strawberry samples. SA concentrations were determined using the microfluidic aptamer-based detection method following sample processing, with additional 10×, 100×, 1000×, and 10,000× dilutions. The shades of red represent strawberry samples subjected to maceration, centrifugation, filtration, and the bead cleaning step, while the shades of brown indicate samples that underwent maceration and the bead cleaning step only, without centrifugation. Error bars represent the ± standard deviation of two replicas.

The microfluidic assay results for SA detection (Figure 5) aligned with HPLC findings, confirming the assay’s reliability. Given that the microfluidic assay is optimised for SA concentrations in the 10^−9^ to 10^−6^ mg/mL range, and the infected strawberry sample contained approximately 10^−3^ mg/mL of SA (as determined by HPLC), dilution was necessary for accurately reaching a concentration within the range of detection of the developed assay analysis. Following the sample treatment protocol (Section 2.3), the infected sample underwent serial dilutions until reaching a concentration within the assay’s sensitivity range. A 1000-fold dilution yielded a fluorescence signal corresponding to an SA concentration of 10^−6^ mg/mL on the response curve, corresponding to a concentration of 10^−3^ mg/mL prior to dilution, matching the results obtained via HPLC and validating the assay’s capacity to detect SA at this concentration.

An alternative sample treatment protocol was evaluated to simplify the process for potential field use, replacing the original method that required centrifugation. This simplified approach involved only maceration followed by a bead cleaning step. However, as shown in Figure 5, while both methods yielded similar results, the simplified protocol was less effective in thoroughly cleaning the sample compared to the centrifugation-based method. This suggests that, although the simplified treatment is more convenient, centrifugation remains essential for optimal sample preparation and analysis.

It’s important to note that the salicylic acid (SA) concentrations reported in the previous sections are five times lower than their actual values. This discrepancy arises from the final step of the sample treatment protocol, which involves a 5× dilution of the samples. Consequently, the estimated SA concentration in the infected samples prior to dilution is approximately 4 × 10^−2^ mg/mL.

## 4. Conclusions

In this study, we developed a microfluidic platform suitable for field applications, incorporating a simplified sample cleaning protocol for the detection of salicylic acid (SA) in plants. SA serves as a biomarker indicative of plant stress, especially biotic stresses such as fungal infections, accumulating at infection sites as part of the plant’s defence mechanisms. Continuous monitoring of SA levels enables early detection of such infections.

The platform utilises an aptamer-based assay, employing streptavidin-coated microbeads to immobilise biotinylated aptamers within the microfluidic channel. Detection is achieved through a labelled DNA strand complementary to a segment of the aptamer. In the presence of the analyte, the aptamer undergoes a conformational change, leading to an increase in the fluorescence signal. Sensitivity evaluations under buffer conditions and validations with strawberry samples infected with *Botrytis cinerea* demonstrated the platform’s capability to detect SA concentrations, with limits of detection ranging from 10^−9^ to 10^−6^ mg/mL.

The platform also features simplified sample treatment protocols, eliminating the need for complex analyte extraction and isolation procedures. An improved protocol, omitting the centrifugation step, is field-ready and does not require sophisticated laboratory equipment; however, it slightly reduces assay sensitivity. The detected concentration range aligns with reported values and the method exhibits greater sensitivity compared to other SA detection techniques. The developed sample treatment protocol, together with the microfluidic assay, allows us to obtain results in under 2 h, surpassing the traditional methods that require lengthy procedures, both at the analyte extraction and the detection stage [9,28]. Additionally, as quantitative information as to how SA values change with infection levels is limited, as baseline values are cultivar dependent, the developed device could be used to measure SA values throughout time and provide valuable information that farmers could use to optimise their crop management.

Utilising this platform in the field allows for rapid, sensitive, and continuous monitoring of SA concentrations in strawberries and other fruits, facilitating timely infection detection. This capability equips farmers with tools to implement preventive measures, effectively controlling the spread of pathogens that could lead to yield losses during harvest, transportation, and storage.

## Figures and Tables

**Table 1:** Sequence of SA aptamer and fDNA molecules.

	Sequence (5’-3’)	Modification 5’ End	Modification 3’ End
SA Aptamer	CTTTCCCTACACGACGCTCTTCCGATCTTTCCCGTTACCCTTATCTCATGCCTGCACTTGATCATGGTCTGTAGGCACCATCAATAGATCG	Biotin	None
fDNA	CGG GAA AGA TCG GAA GAG	Alexa_430_	None

## Data Availability

The raw data supporting the conclusions of this article are available from the authors upon request. All relevant data are also included within the article and its Supplementary Materials.

## Supplementary Materials

The following supporting information can be downloaded at https://www.mdpi.com/article/10.3390/bios15050266/s1, Figure S1: Chromatograms obtained via HPLC for strawberries purchased from a local supermarket: (A) Chromatogram from the strawberry sample; (B) Chromatogram from the strawberry sample spiked with 0.025 mg/mL of SA. In graph (B), where 0.025 mg/mL of SA was spiked, a peak appears at a retention time of 3.12 min. However, in graph (A), no peak is observed at the same retention time. This indicates that the strawberries purchased from a local supermarket do not naturally contain SA, making it an ideal matrix for constructing the calibration curve. Figure S2: A calibration curve was established using High-Performance Liquid Chromatography (HPLC) by analysing samples with known concentrations of salicylic acid (SA). The resulting data was plotted, with the peak area on the y-axis and the corresponding SA concentrations on the x-axis. This calibration curve allowed for the determination of unknown SA concentrations in real samples. Specifically, the infected sample by Botrytis cinerea produced a peak area of 37,875 A.U. By substituting this value into the equation of the calibration line, the SA concentration in the contaminated sample was calculated to be 8 × 10⁻³ mg/mL. This approach utilizes the linear relation-ship between peak area and concentration, providing a precise means of quantifying SA in complex samples. Figure S3: A selection of the results of optimization assays of the concentration of aptamer, and labelled DNA to be used in the aptamer-based assay. The excitation wavelength was set be-tween 450 and 490 nm (blue). Error bars indicate the standard deviation (±) of two replicas. Figure S4: Evaluation of the assay specificity through measurements of buffer solutions spiked with salicylic acid, abscisic acid and jasmonic acid. The excitation wavelength was set between 450 and 490 nm (blue). Error bars indicate the standard deviation (±) of two replicas.
