# Supplementary material for: Aptamer-Based Microfluidic Assay for In-Field Detection of Salicylic Acid in Botrytis cinerea-Infected Strawberries
Source: Biosensors (Basel). 2025 Apr 22;15(5):266. doi: 10.3390/bios15050266 (PMC12109390; doi:10.3390/bios15050266)
Supplement: Supplementary file 1 [file biosensors-15-00266-s001.zip › biosensors-3564291-supplementary.pdf]

Supplementary Information

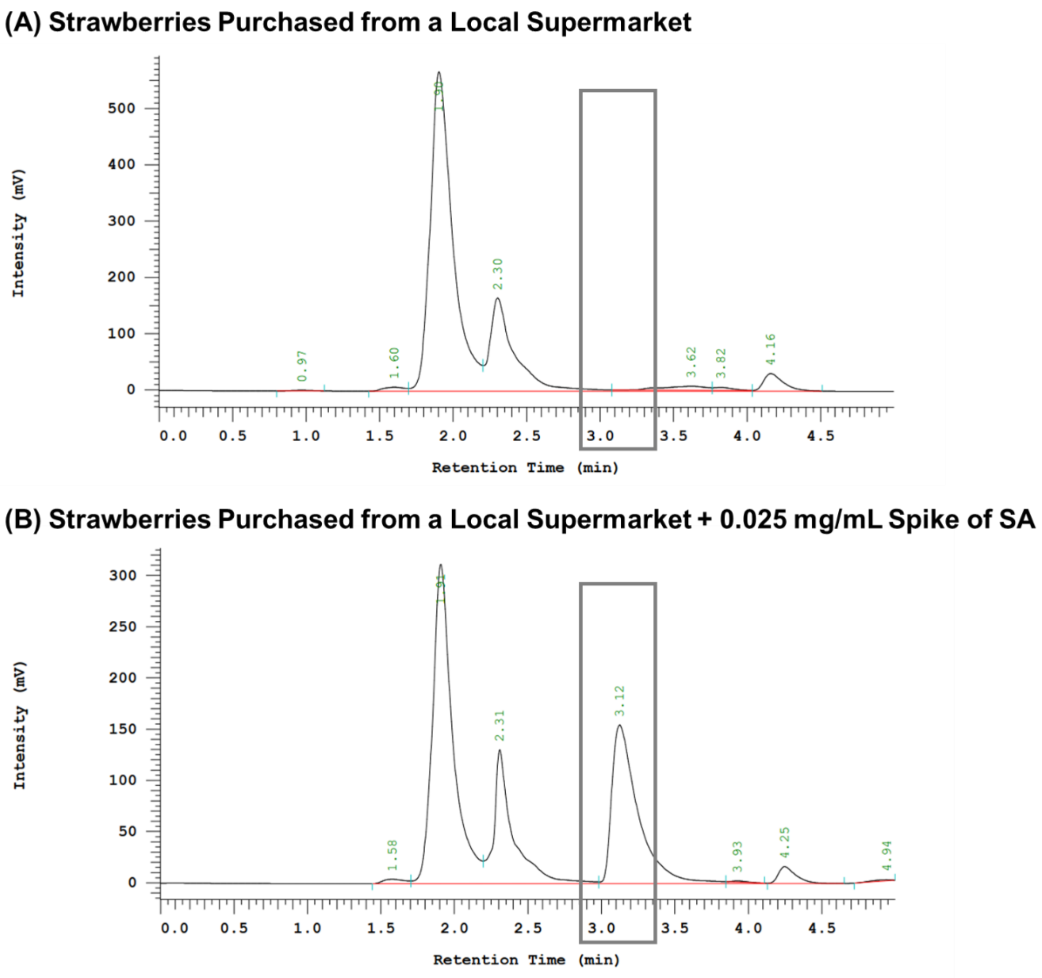

Figure S1. *Chromatograms obtained via HPLC for strawberries purchased from a local supermarket: (A) Chromatogram from the strawberry sample; (B) Chromatogram from the strawberry sample spiked with 0.025 mg/mL of SA. In graph (B), where 0.025 mg/mL of SA was spiked, a peak appears at a retention time of 3.12 minutes. However, in graph (A), no peak is observed at the same retention time. This indicates that the strawberries purchased from a local supermarket do not naturally contain SA, making it an ideal matrix for constructing the calibration curve."*

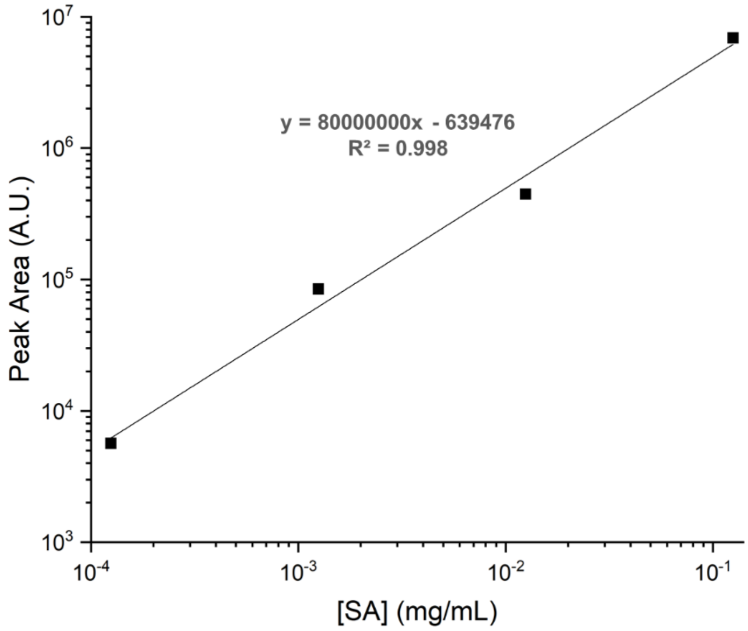

Figure S2. *A calibration curve was established using High-Performance Liquid Chromatography (HPLC) by analysing samples with known concentrations of salicylic acid (SA). The resulting data was plotted, with the peak area on the y-axis and the corresponding SA concentrations on the x-axis. This calibration curve allowed for the determination of unknown SA concentrations in real samples. Specifically, the infected sample by Botrytis cinerea produced a peak area of 37,875 A.U. By substituting this value into the equation of the calibration line, the SA concentration in the contaminated sample was calculated to be* 8×10^{-3} *mg/mL. This approach utilizes the linear relationship between peak area and concentration, providing a precise means of quantifying SA in complex samples.*

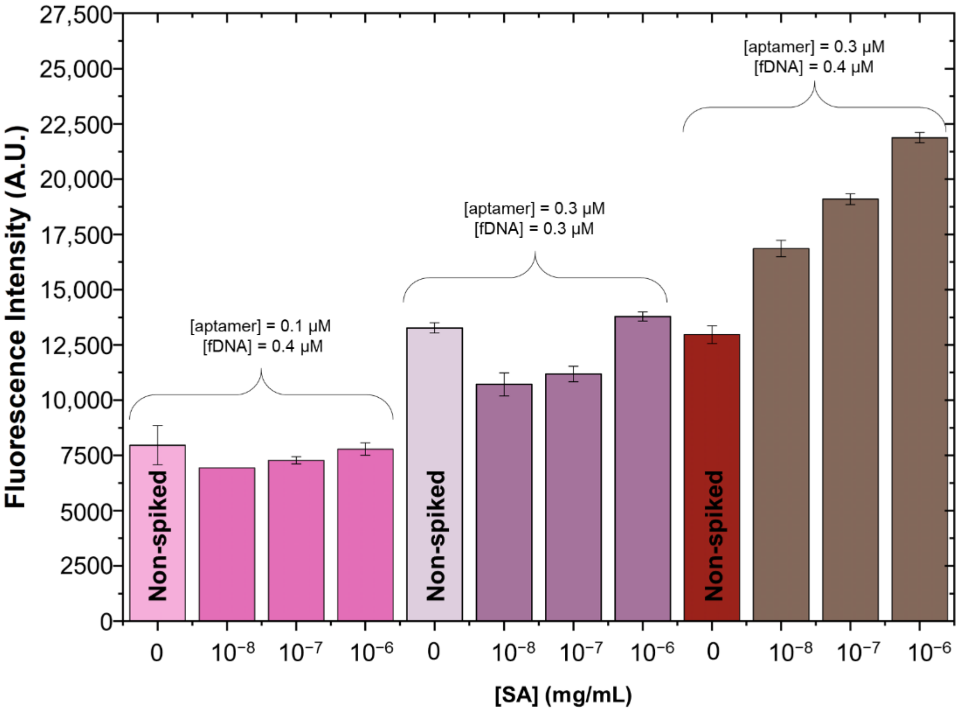

Figure S3. *A selection of the results of optimization assays of the concentration of aptamer, and labelled DNA to be used in the aptamer-based assay. The excitation wavelength was set between 450 and 490 nm (blue). Error bars indicate the standard deviation (±) of two replicas.*

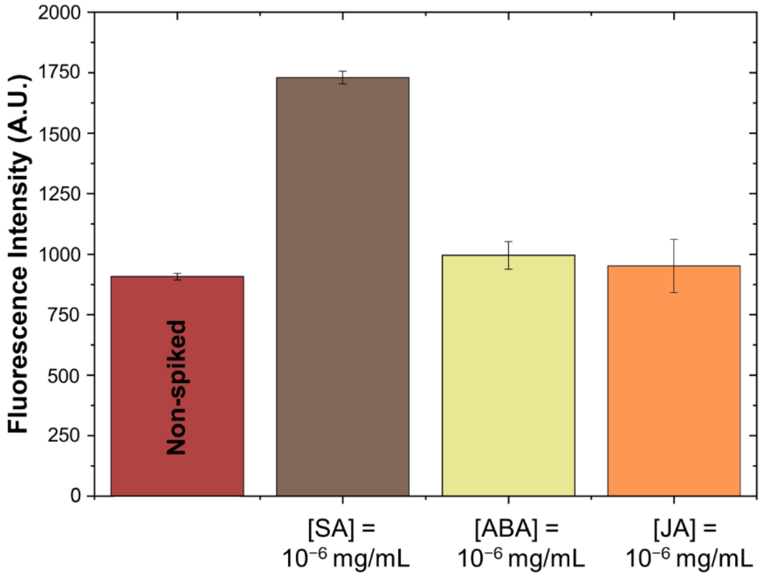

Figure S4. *Evaluation of the assay specificity through measurements of buffer solutions spiked with salicylic acid, abscisic acid and jasmonic acid. The excitation wavelength was set between 450 and 490 nm (blue). Error bars indicate the standard deviation (±) of two replicas.*
